# The role of IL-33 in depression: a systematic review and meta-analysis

**DOI:** 10.3389/fpsyt.2023.1242367

**Published:** 2023-11-01

**Authors:** Renli Liu, Liping Liu, Shiying Ren, Chaojie Wei, Ying Wang, Dong Li, Wenxin Zhang

**Affiliations:** ^1^Department of Clinical Immunology, Xijing Hospital, Fourth Military Medical University, Xi’an, China; ^2^Department of Pathology, The First Hospital of Jilin University, Changchun, China; ^3^Department of Immunology, College of Basic Medical Sciences, Jilin University, Changchun, China

**Keywords:** IL-33, ST2, MDD, BD, microglial cells, depression

## Abstract

Depression has long been considered a disease involving immune hyperactivation. The impact of pro-inflammatory cytokines such as TNF-α, IL-1β, IL-6, and IL-8 on depression has been widely studied. However, the effect of IL-33, another pro-inflammatory cytokine, has been less researched. Currently, research on the correlation between IL-33 and depression risk is inconsistent. In response to these divergent results, we conducted a review and meta-analysis aimed at resolving published research on the correlation between IL-33 and depression risk, and understanding the potential role of IL-33 in the development and treatment of depression. After searching different databases, we analyzed 8 studies. Our meta-analysis showed that IL-33 had a positive correlation with reduced risk of depression. The pooled standard mean differences (SMD) = 0.14, 95% confidence interval (CI): 0.05–0.24. Subgroup analysis results showed that IL-33 and ST2 levels in cerebrospinal fluid and serum is positive correlated with reduced risk of major depressive disorder (MDD) and bipolar disorder (BD). According to the characteristics of the included literature, the results mainly focuses on Caucasian. Furthermore, according to the subgroup analysis of depression-related data sources for disease or treatment, the correlation between IL-33 and depression risk is reflected throughout the entire process of depression development and treatment. Therefore, the change of IL-33 level in serum and cerebrospinal fluid can serve as useful indicators for assessing the risk of depression, and the biomarker provides potential treatment strategies for reducing the burden of the disease.

## 1. Introduction

Depression is a significant problem in modern civilization, and both biological and psychological theories play crucial roles in explaining its etiology (1). Recent studies have shown that excessive activation of the immune system’s inflammation plays an important regulating role in depression (2). Inflammatory markers, including proinflammatory cytokines, anti-inflammatory cytokines, inflammatory enzymes, and oxidative stress, lead to serotonin and melatonin deficiency through the kynurenine pathway, inducing depression (3). Mae (4) proposed the “cytokine-induced depression theory” long ago, speculating on the key role and possible mechanism of inflammatory factors in depression, such as the “inflammation hypothesis, “that acute increases in central and peripheral pro-inflammatory cytokine levels may lead to depressive episodes; and the “neurotrophic factor hypothesis, “that depression episodes are correlated with reduced plasticity of emotion-related brain regions, neuron atrophy, and abnormal nerve cell activity. In recent years, many studies have confirmed the correlation between depression and the central and peripheral concentrations of many pro-inflammatory and anti-inflammatory factors (5, 6), such as IL-6, IL-10, IL-8, IL-1β, IL12p70 etc. Peripheral circulating levels of these factors decrease over time, which is unrelated to disease treatment or clinical response. However, TNF-α levels increase over time.

IL-33 is a cytokine belonging to the IL-1 family and is a pro-inflammatory factor with multiple effects (7). It is expressed in various cell types, including microglia, astrocytes, epithelial cells, and endothelial cells. Meanwhile, its receptor, ST2, is highly expressed in microglia and astrocytes (8). IL-33 can cause and regulate various inflammatory responses (9). A study analyzing the human brain database of the Allen Human Brain Atlas (AHBA) on IL-33 showed that the differential expression of IL-33 in brain areas critical to emotional function, such as the paraventricular nucleus of the hypothalamus, the amygdala, and the cingulate gyrus, is higher than other cytokines commonly examined (such as IL-6 and IL-1β), with no difference in expression in the frontal and middle gyri (10). Anatomically, IL-33 should be a significant cytokine in the development of depression. After inflammatory stimulation, IL-33 is released in substantial quantities by the cells mentioned above, and upon binding with its receptor ST2, activates downstream pathways, altering the expression of pro-inflammatory cytokines, chemokines, and Th2-related cytokines (11). Therefore, from a signal transduction perspective, IL-33 is a crucial aspect of the cytokine theory of depression. Previous research (12) has demonstrated that IL-33 plays a vital role in maintaining synaptic quantity throughout the central nervous system’s development in the brain. The study found that developing astrocytes produce IL-33 and then send signals to microglia, promoting increased synaptic pruning, regulating synaptic remodeling, and quantity to impact brain development (13). This suggests that IL-33 not only affects the development and pathogenesis of depression as a pro-inflammatory factor but also may regulate depression development as a neurotrophic factor.

Currently, there are several studies that have examined the relationship between IL-33 and depression, including changes in the circulating levels of IL-33 during diseases (14, 15) and electroconvulsive therapy for depression over time (16). However, the results of these studies are inconsistent. Therefore, we conducted a meta-analysis of all existing literature on the relationship between IL-33 and depression risk. Our aim was to clarify the specific impact of IL-33 on depression and to contribute a new perspective for the immunological treatment of depression.

## 2. Systematic review

### 2.1. Il-33 regulates the signaling of microglial cells

The regulatory role of IL-33 on microglial phagocytosis is crucial for the pruning and formation of CNS synapses, which has an impact on brain development and emotion-related areas such as the thalamus (17). Microglia are considered target cells of IL-33 (18). It has been shown that IL-33 is a key initiator and regulator of adaptive microglial phagocytosis, migration, activation, and inflammatory response in neurodevelopment and synaptic shaping (8).

IL-33 is a dual-function protein and a member of the IL-1 family of cytokines (19). The N-terminal nuclear domain of IL-33, located in aa1-78 of the protein, has transcriptional inhibitory activity (20). The IL-1-like cytokine domain of IL-33, located in aa111-270, binds to ST2L and exerts pro-inflammatory activity (7). Some studies suggest that degenerative changes in neurodevelopment are an important trigger for the development of depression (12). During early neurodevelopment, the proIL-33 derived from neural glial cells is released and cleaved into its active form by the inflammasome (12), which activates pro-inflammatory cysteine proteases 1 and 5 in the brain (9). Through the IL-33/ST2/AKT signaling pathway, it promotes mitochondrial activity and polarization of microglia into M2 macrophages, which facilitates synaptic remodeling and quantity and helps prevent depression and neurodegenerative diseases (21). IL-33 modulates the activation and polarization of microglia, which may be an indirect evidence that IL-33 suppresses the development of depression. Additionally, IL-33 with its receptor ST2 and IL1RAcP formed a heterodimeric receptor complex which promotes the recruitment of scaffold protein PSD-95 and increases the expression of myocyte enhancer factor 2 C (MEF2C), facilitating the formation of functional excitatory synapses in the hippocampal CA1 neurons and enhancing neural transmission (10). Furthermore, upregulating Pten and Inpp5d can inhibit AKT activity, causing microglia to transition into metabolic quiescence (8). In an LPS-induced anxiety model, IL-33 was upregulated in the basolateral amygdala. Research has shown that IL-33 regulates anxiety by releasing IL-33 from astrocytes in the amygdala, which, through the IL-33/ST2/NF-κB pathway, inhibits the transmission of and GABAergic neurons, and activates the anti-anxiety circuit (13). However, some studies also indicate that IL-33 can inhibit the production of brain-derived neurotrophic factor (BDNF) through the NF-κB pathway (9). IL-33 functions as a transcriptional regulation factor in nuclear to regulate inflammation as well (22), but the exact mechanism is still unclear.

### 2.2. Regulatory effects of IL-33 on depression

The brain and spinal cord are the organs with the highest levels of IL-33 mRNA in the body (23). IL-33 is expressed in endothelial cells, microglia cells, and astrocytes, while its receptor ST2 is mainly located on microglia cells and astrocytes (24). The expression of IL-33 mRNA in microglia cells in the paraventricular nucleus of the hypothalamus and prefrontal cortex is stimulated by acute stress and inflammation induction (25). Moreover, IL-33 mRNA’s expression is upregulated in neurons and astrocytes following subarachnoid hemorrhage (26). External stress, emotional stress, and internal inflammatory stimuli can lead to upregulation of IL-33 expression in the brain, as inflammatory stimuli such as PAMPs (LPS, dsRNA, PAMPs + ATP) induce the production of pro-inflammatory cytokines by microglial cells and mast cells (27). Studies have shown that cytokines can influence the pathogenesis of depression through changes in biogenic amine metabolism in the midbrain nucleus such as dopamine, norepinephrine, and serotonin (28). Stress responses, inflammation stimulation, enhance the activity of the hypothalamic–pituitary–adrenal axis (HPA axis), leading to an increase in the level of cortisol, promoting the increase of inflammatory cytokines such as IL-33, and downregulating neurotrophic factors such as monoamines, DNA methylation, and histone acetylation (29). IL-33 is closely related to peripheral and central immune activation. IL-33 mainly affects the reshaping and quantity of synapses in microglia and astrocytes by binding with ST2, thereby influencing the development of emotion-related brain regions and ultimately affecting the risk of depression (19). Overall, IL-33 has a unique position in depression and can be an essential link in the cytokine theory of depression.

**Figure 1:**
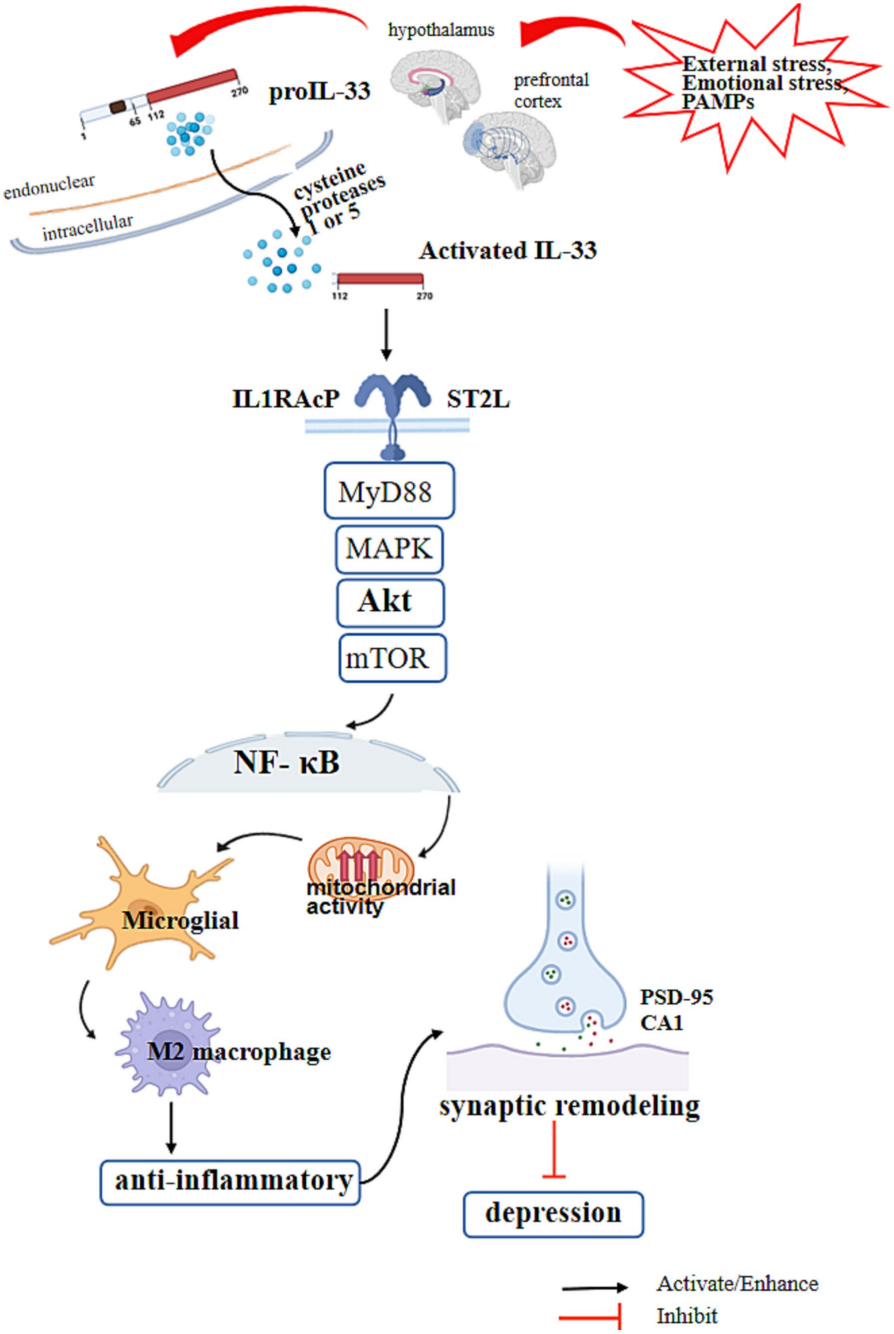
IL-33 regulates the signaling of microglial cells and depression. The N-terminal nuclear domain of IL-33 is located at amino acids 1–78 of the IL-33 protein and has transcriptional inhibition activity. The IL-1-like cytokine domain of IL-33 is located at amino acids 111–270 and has inflammatory activity by binding to ST2L. External stress, emotional stress, and inflammation stimulation (PAMPs) in the body increase the release of IL-33 in microglial cells in the paraventricular nucleus of the hypothalamus and prefrontal cortex. After precursor IL-33 is released, it is cleaved into its active form by cysteine proteases 1 and 5, which are activated by inflammasomes in the brain. IL-33 with its receptor ST2 and IL1RAcP formed a heterodimeric receptor complex which activates downstream Myd88 and MAPK, activates mTOR through AKT, activates NF-κB in the nucleus, promotes microglial mitochondrial activity, and polarizes microglia into M2 macrophages. At the same time, it activates the synaptic recruitment of scaffold protein PSD-95 and the expression of muscle enhancer factor 2C (MEF2C), promotes the formation of functional excitatory synapses in hippocampal CA1 neurons, increases neural transmission, promotes synaptic remodeling and numbers, and reduces the risk of depression.

While no studies have directly assessed the roles of IL-33 in models of chronic stress and depression, our theoretical hypothesis suggests an indirect interaction between human IL-33, microglia in the human brain, and depression. It is known that microglia secrete IL-33 and are regulated by IL-33, which in turn regulates neurodevelopment, synapse number, and reduces depression triggers. This regulation occurs through signaling pathways that influence cell polarization and metabolism. Figure 1 provides a summary of this theoretical relationship. However, further research is needed to gather more robust evidence.

### 2.3. In the cerebrospinal fluid, IL-33 is related to depression

As IL-33 is a large molecule, it faces difficulty in passing through the blood–brain barrier. Furthermore, the cytokine balance between cerebrospinal fluid and serum has not been established, which makes it challenging to determine the effects of IL-33 consistently in depression.

In the brain, IL-33 is secreted by microglial cells and regulates synaptic remodeling (30). It is present in some brain regions that are associated with memory and emotion, thereby influencing depression by regulating the central nervous system’s development (31). Studies indicate that the levels of IL-33 increase in the cerebrospinal fluid of patients with bipolar disorder (BD) (32) and pregnant women with major depressive disorder (MDD) (33). Evidence also suggests that IL-33 in cerebrospinal fluid is related to postpartum depression and Alzheimer’s disease (18). One possible mechanism for this relationship is the induction of tryptophan-2,3-dioxygenase expression by IL-33 (34). This results in the degradation of tryptophan into kynurenine and quinolinic acid metabolites. Since tryptophan is a precursor to serotonin, which is crucial for emotional stability, the metabolites kynurenine and quinolinic acid are known to independently induce depressive symptoms (34). However, there is no clarification of the dose–response relationship between cerebrospinal fluid IL-33 and depression, and further research is necessary. Research indicates that serum IL-33 is not associated with prenatal or perinatal depression. However, several previous studies have demonstrated that increased levels of inflammatory cytokines including IL-33 in urine (35) and serum (36, 37) may increase the risk of postpartum depression. These findings support the potential risk of depression associated with IL-33 in the cerebrospinal fluid in related diseases.

### 2.4. In the serum, IL-33 is associated with depression

However, some people also believe that although the cytokine IL-33 is a large molecule, there are multiple mechanisms in the body that promote transportation between the cerebrospinal fluid and blood plasma, such as leakage areas of the blood–brain barrier; active transport of molecules; activation of cells around the vascular system; and binding of cytokine receptors with incoming nerve fibers (38). The HPA axis is an important pathway for coordinating the release of inflammatory cytokines from immune cells in the periphery and central nervous system, and may regulate changes in central and peripheral IL-33 levels and expression activity (39). There is evidence that serum IL-33 is directly related to depression associated with systemic lupus erythematosus (SLE) (40) and alopecia areata (AA) (41). Furthermore, women with a history of child abuse have higher levels of IL-33 monomer protective genes in their serum. Higher levels of IL-33 have also been detected in the serum of patients with recurrent MDD compared to those with single episode MDD and no MDD (42).

### 2.5. The impact of IL-33 in tDCS and depression

Transcranial direct current stimulation (tDCS) has gradually become an important treatment modality for depression (especially MDD) in addition to medication (16). Studies have shown that serum levels of proinflammatory cytokines decrease as tDCS treatment progresses (43). IL-33, as a classic proinflammatory factor and abundant in the brain’s emotion regulation center, is speculated to participate in the regulation of tDCS treatment for depression. However, current studies did not indicate the association between IL-33 and tDCS treatment, and there are currently no relevant literature linking IL-33 with tDCS, other electrical current therapy, and antidepressant drugs. Therefore, further research is needed on this topic.

### 2.6. IL-33 SNPs and depression

The SNP alleles rs11792633 and rs7044343 in the *IL-33* gene regulate the association between childhood abuse history in females and recurrent major depressive disorder (rMDD) (42). Compared to females with a history of childhood abuse and no MDD or single episode major depressive disorder (sMDD), females with rMDD and a history of childhood abuse have a higher *IL-33* SNP protective genotype in their serum. This protective genotype is not different among females with sMDD and no MDD which without a history of childhood abuse. This indicates that the SNP alleles rs11792633 and rs7044343 in the *IL-33* gene are associated with a decreased occurrence of rMDD in females with a history of childhood abuse. However, the precise mechanism by which these alleles influence the IL-33 protein signaling and impact the risk of rMDD is still not well-understood, and further research is needed.

## 3. Il-33 and the risk of depression: a meta-analysis

Due to the inconsistency in results of IL-33 levels in depression research, we have summarized and consolidated all IL-33 and depression-related studies in the hope of finding potential pathological and physiological influences of IL-33 on the development of depression, thus adding a new chapter to clinical interventions for depression.

### 3.1. Study characteristics

**Figure 2:**
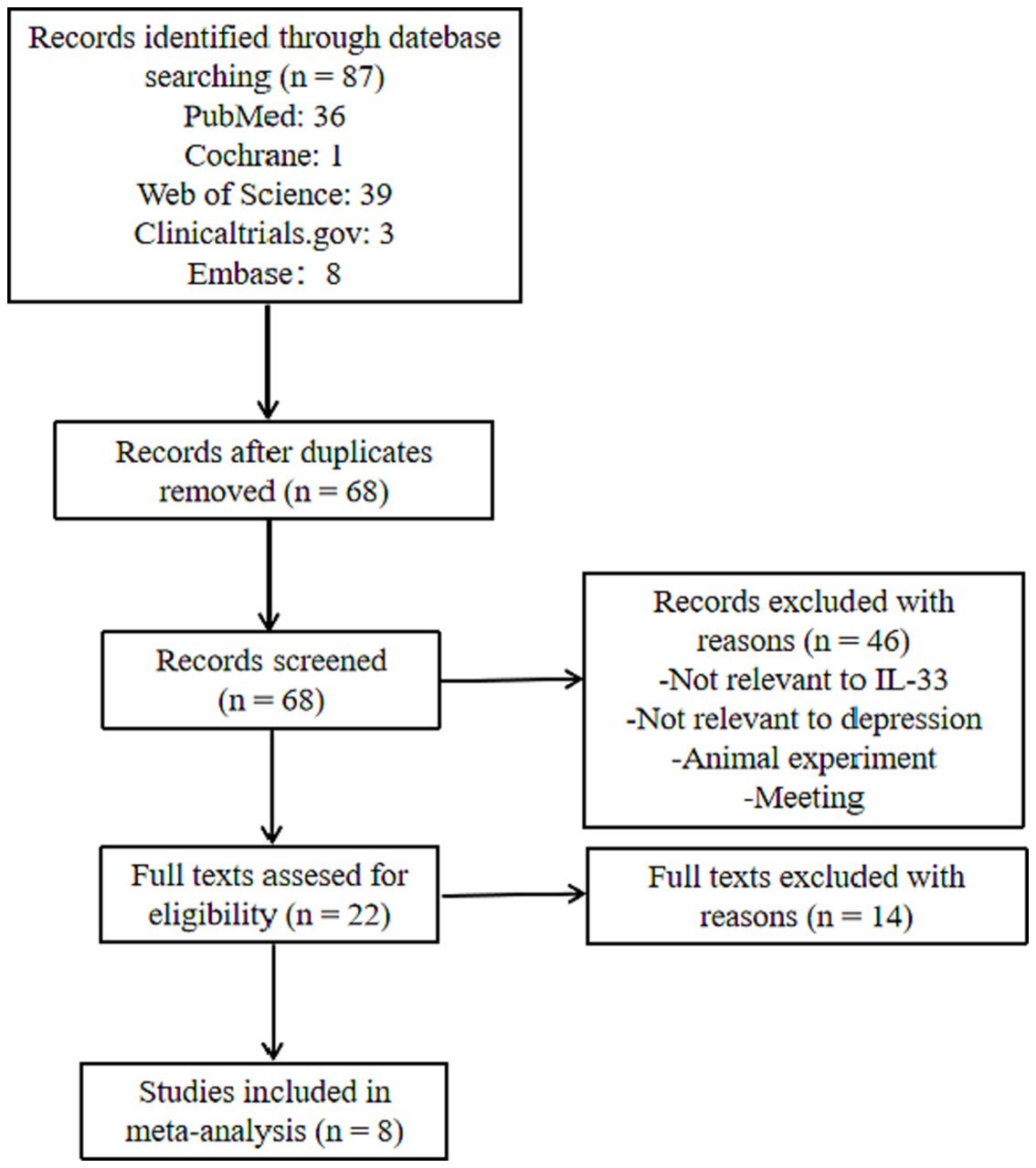
Flow chart of study selection.

We searched for all articles on IL-33 and depression, published before April 1, 2023, without any language restrictions, by using the keywords and Mesh terms IL-33, ST2, MDD, BD, depression, neuro, and searching on PubMed, Web of Science, Embase, Cochrane Library, and Clinicaltrials.gov websites. The literature screening process is shown in Figure 2. We found a total of 87 articles related to IL-33 and depression, and upon removing duplicates, we selected articles that met the following criteria: (1) all studies must be case–control or clinical cohort studies that include the relationship between IL-33 and susceptibility to depression; (2) all patients must fulfill the diagnostic criteria for depression or bipolar disorder; (3) the study must comprise mean and SD or OR/RR/HR and 95% CI of IL-33 in cerebrospinal fluid or serum; and (4) the study population must be human. Studies that did not meet the inclusion criteria were excluded. Finally, we included 8 studies that satisfied the data inclusion criteria (16, 32–34, 40–43). Two researchers screened these articles continuously, and after two screenings, the results were consistent. The detailed information regarding included studies is shown in Table 1.

**Table 1:** Characteristics of the studies included.

Study	Diagnostic criteria	Type of depression	Sample size		Relative gene	Source of cytokine	Cytokine measurement methods	Ethnicity	Significance	Relative disease
			Case	Control						
Mak et al. (40)	HADS	MDD	54	54	IL-33	Serum	ELISA	Sinensis, Australis, Others	Yes	SLE
Kudinova et al. (42)	DSM-IV	MDD	10	20	IL-33	Serum	ELISA	Caucasian	Yes	Depression
Brunoni et al. (16)	HDRS-17	MDD	236	214	IL-33	Serum	ELISA	Caucasian	No	tDCS
Miller et al. (34)	IDS-SR30	MDD	91	26	IL-33	Cerebrospinal fluid	FlowJ0	Caucasian	Yes	Depression
Brunoni et al. (33)	HDRS-17	BD	59	245	IL-33/ST2	Cerebrospinal fluid	ELISA	Caucasian	Yes	Depression
Bain et al. (41)	HADS	Depression and Anxiety	39	26	IL-33	Serum	U-plex multiplex assay platforms	Caucasian	Yes	AA
Bavaresco et al. (32)	BRIAN	BD	36	46	IL-33	Serum	ELISA	Caucasian	No	Depression
Goerigk et al. (43)	HDRS-17	BD	26	26	IL-33/ST2	Serum	ELISA	Caucasian	No	tDCS

MDD, major depressive disorder; BD, bipolar disorder; SLE, systemic lupus erythematosus; AA, alopecia areata; tDCS, transcranial direct current stimulation.

### 3.2. Meta-analysis

**Figure 3:**
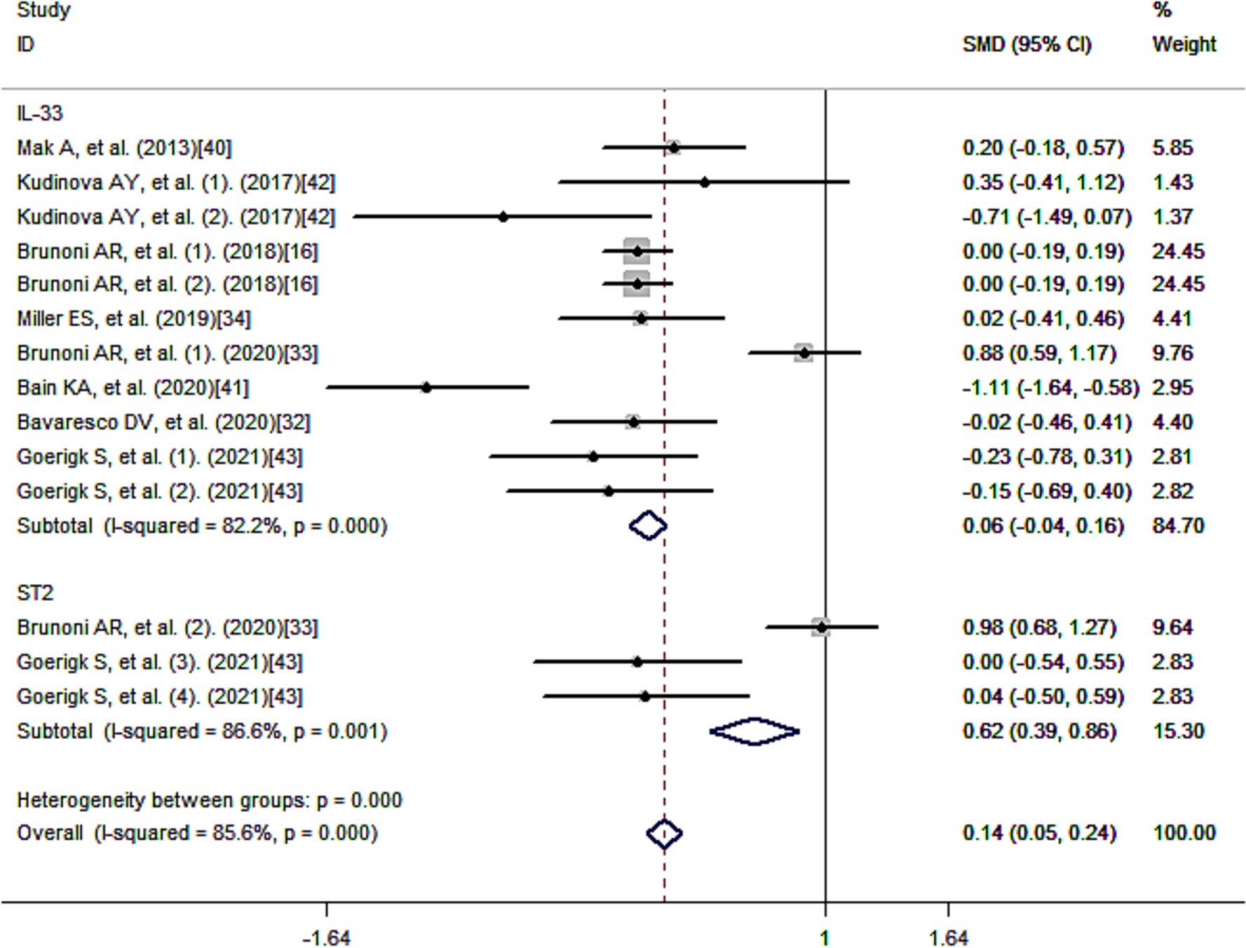
Meta-analysis of IL-33 and the risk of depression.

Figure 3 presents the standard mean differences (SMD) of IL-33 after multivariate adjustment. Meta-analysis revealed a positive correlation between IL-33 and reduced risk of depression, suggesting a protective effect of IL-33 against depression. The pooled SMD was 0.14 [95% confidence interval (CI): 0.05–0.24], and there was considerable heterogeneity among the studies (I^2^ = 85.6%; *p* < 0.001).

### 3.3. Subgroup analysis

**Figure 4:**
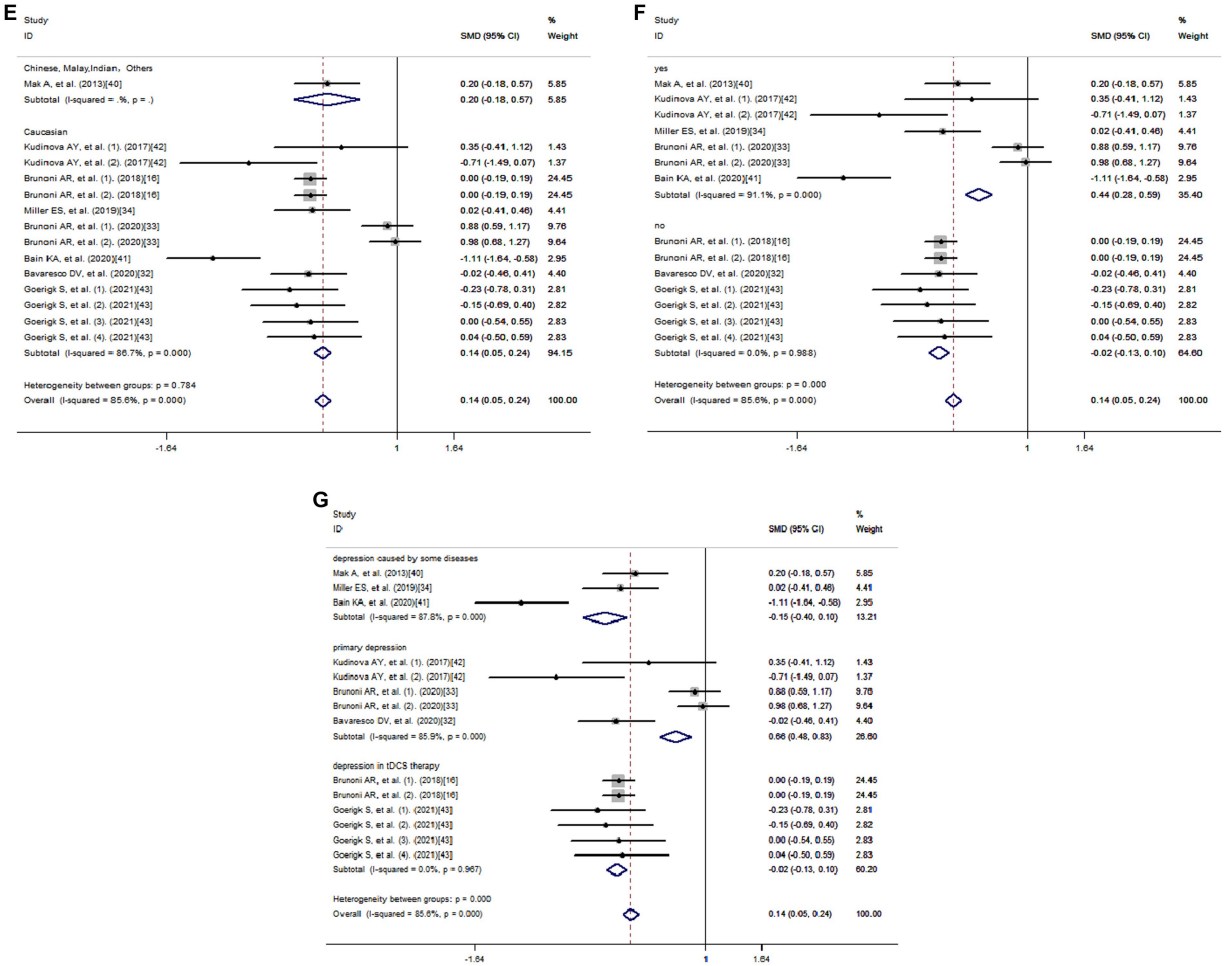
Subgroup analysis of meta-analysis. **(A)** Genes. **(B)** Depression types. **(C)** Sources of cytokines. **(D)** Cytokine measurement methods. **(E)** Ethnicity. **(F)** Significance. **(G)** Data Sources.

Using a method of sub-analysis, this study investigates the impact of potential factors on the correlation between IL-33 and depression risk (Figure 4). The sub-analysis of genes shows that both *IL-33* (SMD = 0.06, 95% CI: −0.04-0.16; I^2^ = 82.2%; *p* = 0.000) and its receptor *ST2* (SMD = 0.62, 95% CI: 0.39–0.86; I^2^ = 86.6%; *p* = 0.001) are associated with decreased risk of depression (Figure 4A); the sub-analysis of depression types, including MDD (SMD = 0.01, 95% CI: −0.10-0.13; I^2^ = 0.2%; *p* = 0.415) and BD (SMD = 0.48, 95% CI: 0.33–0.64; I^2^ = 85.1%; *p* = 0.000), indicates that IL-33 and ST2 are protective factors for both types of depression (Figure 4B); the sub-analysis of sources of cytokines demonstrates that IL-33 and ST2 from serum (SMD = −0.05, 95% CI: −0.15-0.06; I^2^ = 54.5%; *p* = 0.015) and cerebrospinal fluid (SMD = 0.76, 95% CI: 0.57–0.95; I^2^ = 85.3%; *p* = 0.001) are both positively correlated with reduced depression risk (Figure 4C); the sub-analysis of cytokine measurement methods shows that various techniques, such as ELISA (SMD = 0.19, 95% CI: 0.10–0.29; I^2^ = 83.7%; *p* = 0.000), FlowJo and U-plex multiplex assay platforms, do not affect the relationship between cytokines and decreased risk of depression (Figure 4D); the sub-analysis of ethnicity reveals that IL-33 and ST2 are positively associated with reduced depression risk in Caucasian (SMD = 0.14, 95% CI: 0.05–0.24; I^2^ = 86.7%; *p* = 0.000) (Figure 4E); due to limited literature, sub-analysis of other ethnicity groups was not possible. Most of the literature collected showed that IL-33 and ST2 are correlated with depression (33, 34, 40–42), while some studies found no statistical significance between IL-33 or ST2 levels and depression (16, 32, 43). When we summarized these non-significant findings, we still observed a positive association between IL-33 or ST2 and reduced depression risk (SMD = −0.02, 95% CI: −0.13-0.10; I^2^ = 0.0%; *p* = 0.988) (Figure 4F). Additionally, sub-analysis of the sources of depression-related data shows that IL-33 or ST2 are protective factors in depression in tDCS therapy (SMD = −0.02, 95% CI: −0.13-0.10; I^2^ = 0.0%; *p* = 0.967), primary depression (SMD = 0.66, 95% CI: 0.48–0.83; I^2^ = 85.9%; *p* = 0.000), depression caused by some diseases (SMD = −0.15, 95% CI: −0.40–0.10; I^2^ = 87.7%; *p* = 0.000) (Figure 4G).

All SMDs in the studies were adjusted based on high-risk factors for depression, such as median annual income, age, female sex, body mass index, smoking status, years of schooling, and some clinical characteristics, including hypertension, diabetes, physical activity. At the same time, all studies included a placebo control group to ensure that the observation results were stable over time and to exclude the possibility of natural disease fluctuations affecting the results.

### 3.4. Meta-regression

**Figure 5:**
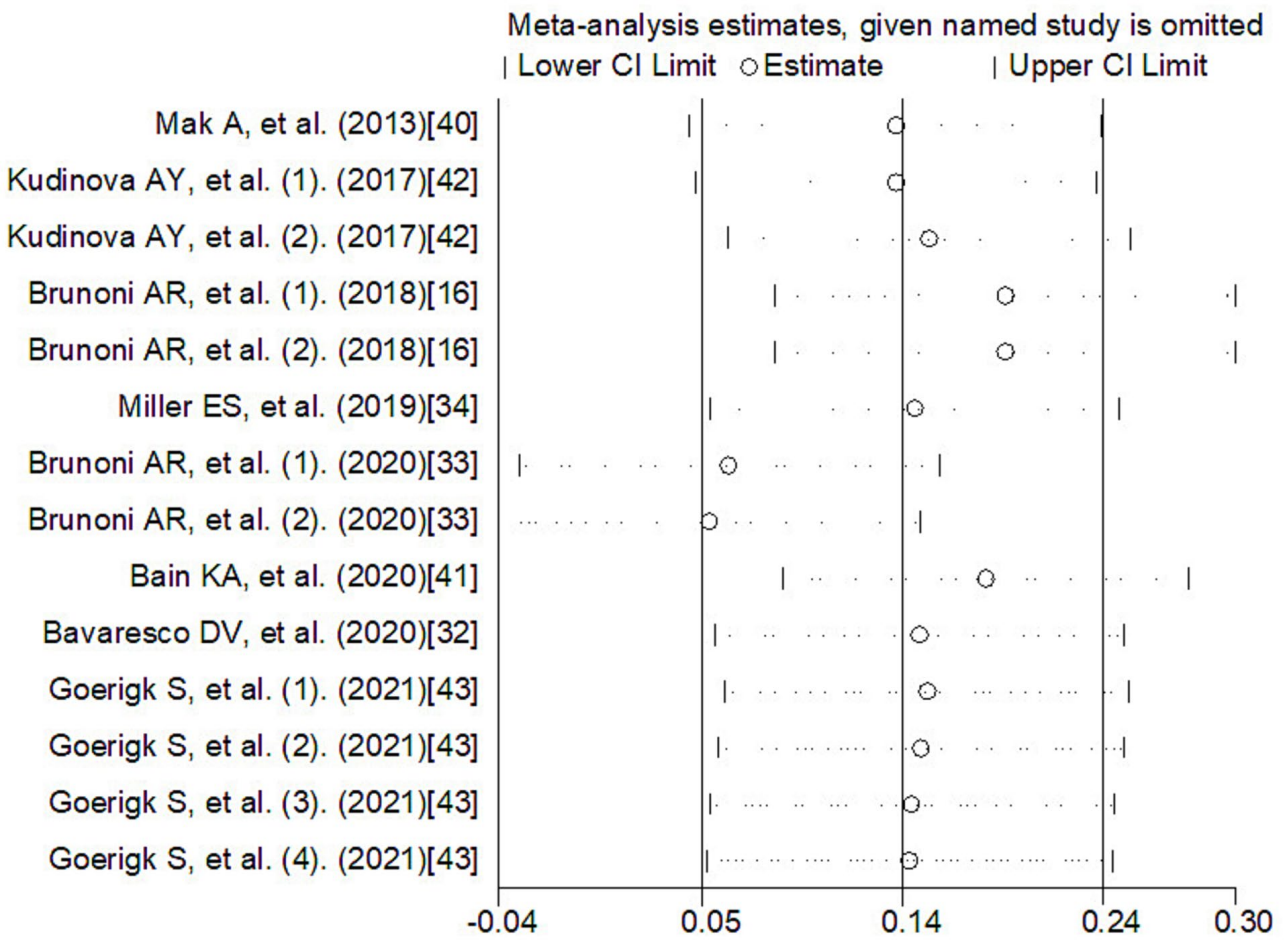
The sensitivity analysis of meta-analysis. CI, confidence interval.

**Figure 6:**
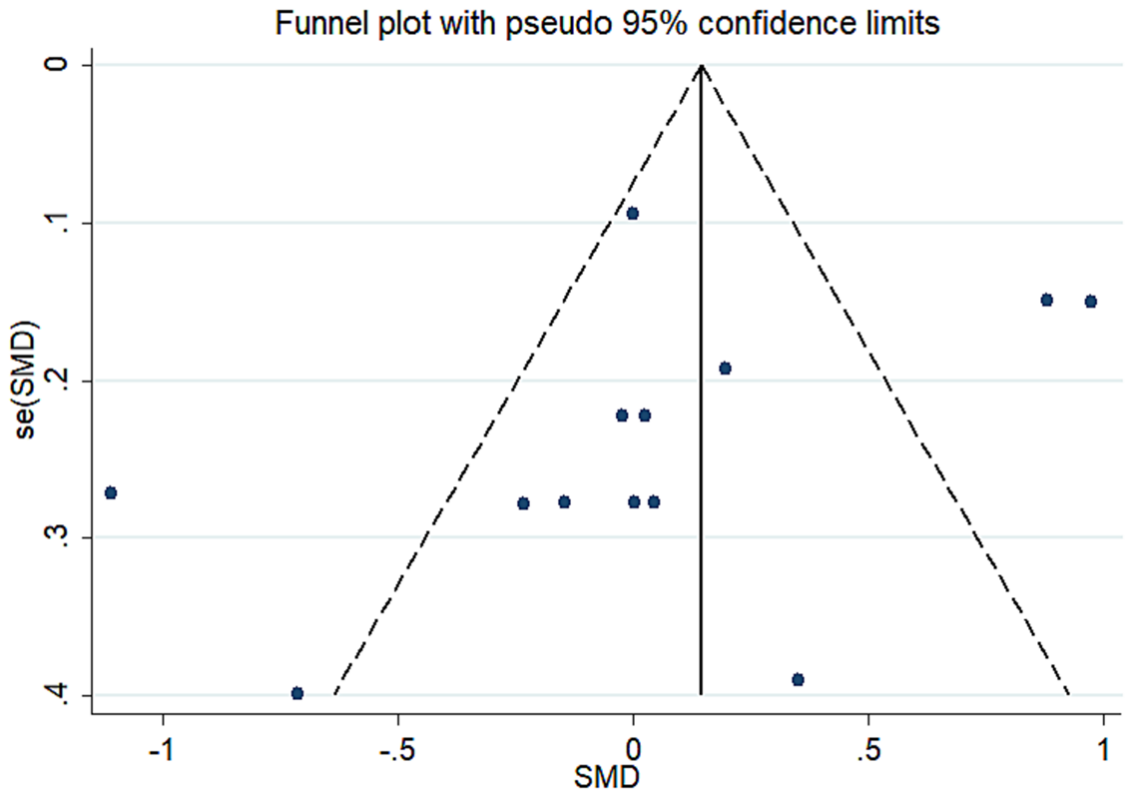
Funnel plot of the meta-analysis. se, standard error; SMD, the standard mean differences.

Meta-regression was used to investigate the sources of heterogeneity, using genes, types of depression, source of cytokine, cytokine measurement methods, ethnicity, and data sources as covariates (Table 2). The results showed that genes, type of depression, ethnicity, and significance may be significant covariates of heterogeneity among studies. The sensitivity analysis results showed that Brunoni et al. (16) may be the main source of heterogeneity (Figure 5). Funnel plots (Figure 6), Begg test, and Egger test were used to assess publication bias. However, no publication bias was found (Begg test *p* = 0.547; Egger test *p* = 0.492).

**Table 2:** Meta-regression based on genes, types of depression, source of cytokine, cytokine measurement methods, ethnicity, significance, data sources as covariates of depression disease.

Covariate	*p*
Genes	0.235
Types of depression	0.544
Source of cytokine	0.009
Cytokine measurement methods	0.038
Ethnicity	0.779
Significance	0.510
Data sources	0.023

## 4. Discussion

The effect of IL-33 on depression has been increasingly focused on, but the existing research results are not consistent. We conducted a meta-analysis of all the current studies on differences in IL-33 levels and depression risk, which showed a protective effect of IL-33 against depression. Additionally, IL-33 can result in a lower recurrence rate of electroconvulsive therapy for MDD and BD. In addition to the result that an increase in circulating IL-33 levels decreases the risk of depression (44), related studies also indicated that certain SNP haplotypes in the *IL-33* gene, such as rs11792633 and rs7044343, directly protect against the occurrence of depression, but these two haplotypes are only associated with rMDD in women who have experienced childhood abuse (42). The connection between other SNPs of *IL-33* and depression has not yet been discovered and needs further research. Degenerative changes in neurodevelopment are an important cause of depression. In addition to IL-33 having an impact on depression with regards to differences in levels as a pro-inflammatory factor (45), a study found that IL-33 also acts as a neurotrophic factor, affecting the remodeling and quantity of brain neural synapses to prevent the onset of depression (13). IL-33 is produced by developing microglia cells and, under normal physiological conditions, it signals to promote mitochondrial metabolism and polarization towards M2-type macrophages in microglia cells. It also facilitates synaptic phagocytosis and remodeling in microglia cells. These data suggest that IL-33 is necessary for maintaining normal synaptic numbers and neurodevelopment in the spinal cord and thalamus. Since ST2 is the receptor for IL-33, the biological and immunomodulatory effects of the cytokine IL-33 are achieved through binding to its receptor ST2, activating downstream pathways and nuclear factors, and thus playing a role in the development of diseases. Certain studies have also demonstrated a correlation between circulating ST2 levels and depression (46). Therefore, we also included changes in circulating ST2 levels in our study, which showed a positive correlation between changes in ST2 levels and an decreased risk of depression, consistent with the effect of IL-33 on depression. This is consistent with the theoretical hypothesis results in the systematic review section. However, no studies have yet discovered a correlation between *ST2*-related SNPs and depression. An experimental study was conducted on male mice to investigate the role of IL-33 in the brain (47). The findings suggest that chronic unpredictable mild stress (CUMS) and social stress (SS) may lead to anxiety or depression-like behavior, whereas restraint stress (RS) appears to reduce depression-like behavior. The levels of IL-33 in the frontal cortex and hippocampus were assessed using one-way or Brown Forsythe ANOVA in the RS, CUMS, SS, and control groups. However, the results seem not show statistically significant differences. Nevertheless, this study is of great significance as it discovered that behavioral and endocrine factors, as well as central gene expression, support the involvement of the HPA axis, neuroinflammation (specifically IL-33, s100a8, Tnfrsf1a), monoamines, epinephrine, and adipokine signaling in anxiety or depression-like behavior. These findings provide new evidence regarding the role of IL-33 in modulating the development of depression.

Previous research has indicated that there are different immune patterns in the immune-inflammatory response system (IRS) and compensatory immune regulatory response system (CIRS) in individuals with BD and MDD (48). IL-33 or ST2 primarily regulate the IRS and CIRS in BD (49), but no significant regulatory effect has been detected in MDD. Thus, we conducted a subgroup analysis of the two types of depression and found that both BD and MDD were protected by IL-33 or ST2 levels. This protection may be linked to the fact that IL-33 is a pro-inflammatory factor with complex biological activity and function. Even though our study only included humans, the cross-species effects of IL-33 in depression have been validated (49). MDD and coronary artery disease (CAD) commonly coexist, wherein depression elevates the likelihood of cardiovascular disease development and detrimentally affects the course of cardiovascular disease (50, 51). A recent study on mouse depression showed (49) that the IL-33 gene is associated with mouse social stress (SS), and changes in mouse coronary blood flow, and that there is a link between MDD and coronary heart disease. Our prior study demonstrated that IL-33’s immune regulatory role in CAD regulation is inconsistent, which may be due to the different biological roles of IL-33 as a cytokine and transcription factor (52). Hence, we hypothesize that IL-33’s biological activity in acting as both a cytokine and transcription factor can influence the regulation of depression.

From an anatomical perspective, it has been found that IL-33 is abundant in human brain regions that are crucial for emotional function (53), and its expression is significantly higher than that of other pro-inflammatory cytokines (54). Therefore, many studies have focused on changes in IL-33 levels in cerebrospinal fluid. The signal transduction of IL-33 and the HPA axis may regulate circulating IL-33 levels, but there is no statistical significance regarding the correlation between serum IL-33 levels depression in some studies. We analyzed all studies related to the correlation between serum IL-33 levels and depression, and the results showed that serum IL-33 or ST2 levels are associated with a reduced risk of depression.

Some of the included research results show no significance between IL-33 or ST2 and depression. However, we believe that some of the included depression patients may have taken antidepressant medication during the study period (32). The impact of these drugs on circulating IL-33 or ST2 was not studied or explained, while the control group was not affected by these drugs. This may be a possible reason for the false negative correlation between IL-33 or ST2 and depression.

Currently, the most commonly used method for measuring cytokines is ELISA, followed by the FlowJo and U-plex multiplex assay platforms. However, it appears that all cytokine measurement methods do not affect the analysis results.

Due to the inclusion of 8 studies, 7 of which were focused on Caucasian, and the other study included Asians, mixed race, and other races, subgroup analysis could not be performed. We only conducted subgroup analysis on Caucasian, and the subgroup analysis results were consistent with the overall results.

Due to the fact that the research we included is not solely focused on primary depression but also includes depression caused by SLE, AA, postpartum depression, and other conditions. Additionally, some of the data pertains to changes in IL-33 or ST2 baseline levels or changes after 3 or 6 weeks of tDCS treatment for depression (43). To investigate the potential differences between the relationship in depression in tDCS therapy and depression caused by some diseases with IL-33 and the relationship in primary depression with IL-33, we conducted subgroup analyses based on the source of data on depression or treatment. The results show that there is no phenomenon of altered outcomes due to non-primary depression, indicating that depression from any source is protected by IL-33. Furthermore, the immune regulation of depression by IL-33 runs through the occurrence, development, and treatment of depression.

The heterogeneity among studies directly influences the interpretation of meta-analysis results (55). Therefore, exploring the potential sources of heterogeneity among studies is an important aspect of this research (56). Our Meta-Regression results show that gene, type of depression, ethnicity, and significance may be significant covariates that affect heterogeneity among studies. Sensitivity analysis results show that Brunoni et al. (33) may be the main source of heterogeneity. Age and gender are important covariates of depression occurrence (57), but only some studies provide relevant data. Due to insufficient information, separate analysis was not carried out, but all studies indicated that these data were obtained by adjusting for depression-related risk factors, and all studies included control groups, which would reduce the impact of covariates such as age and gender on the results. Due to the studies about diagnosis of the IL-33 concentration was not adequate for the diagnostic test, the diagnostic test for depression was not achieved. Although our study has some limitations, it is the first meta-analysis to explore the correlation between IL-33 levels and depression risk. Funnel plots, Begg’s test, and Egg’s test did not find publication bias.

The role of IL-33 in depression plays a crucial role in both the diagnosis and treatment of the disorder. Regarding diagnosis, detecting IL-33 levels in the serum or cerebrospinal fluid of depressed patients can serve as a valuable indicator for disease onset, activity, prognosis, and even the risk of developing depression in individuals with certain primary disorders such as SLE, AA, perinatal conditions, and others. In terms of genotype, detecting SNP haplotypes rs11792633 and rs7044343 in the IL-33 gene of women with a history of child abuse could provide a potentially useful diagnostic marker for identifying a high-risk group for developing rMDD. Moreover, the activation of the IL-33/ST2/NF-KB pathway, along with the release of IL-33 by microglia, shows promise as a novel therapeutic approach for treating depression. When low levels of IL-33 or the presence of SNP haplotypes for genes rs11792633 and rs7044343 are detected, it is crucial to remain vigilant for potential depression in patients. In such cases, providing intensive humanistic care, including counseling, and considering drug treatment in severe cases, becomes essential.

## 5. Conclusion

Through the IL-33/ST3/NF-KB pathway, IL-33 promotes the polarization of microglia towards M2 macrophages, enhances synaptic remodeling and increases synapse numbers, inhibits GABA conduction, prevents depression-inducing factors, and lowers the risk of depression. Our meta-analysis results showed that there is a positive correlation between the levels of IL-33 or ST2 from cerebrospinal fluid and serum and the reduced risk of MDD and BD. IL-33 or ST2 has a protective effect in the occurrence and development of MDD and BD. Based on the characteristics of the included literature, this research results are mainly focused on Caucasians. According to the subgroup analysis of the disease or treatment sources of depression-related data, the correlation between IL-33 or ST2 and depression is reflected in the entire process of the occurrence and development of depression and treatment. Therefore, the levels of IL-33 or ST2 from cerebrospinal fluid and serum can be used as useful indicators to influence the risk of depression. SNP haplotypes of *IL-33*, rs11792633 and rs7044343 can be useful diagnostic and treatment indicators for early depression in women with a history of child abuse. These biomarkers provide potential therapeutic strategies for reducing the burden of disease.

## Data availability statement

The original contributions presented in the study are included in the article/supplementary material, further inquiries can be directed to the corresponding author.

## Author contributions

RL: conceiving the research. RL and LL: data analysis and manuscript writing. SR and CW: data analysis. YW and DL: screening of studies. WZ: manuscript review. All authors contributed to the article and approved the submitted version.
